# Burnout Syndrome Among Medical Practitioners Across India: A Questionnaire-Based Survey

**DOI:** 10.7759/cureus.771

**Published:** 2016-09-08

**Authors:** Deepak Langade, Pranav D Modi, Yazad F Sidhwa, Namita A Hishikar, Amit S Gharpure, Kalpana Wankhade, Jayshree Langade, Kedar Joshi

**Affiliations:** 1 Head, Dept. of Pharmacology, Bharati Vidyapeeth Deemed University Dental College & Hospital, Navi Mumbai; 2 Trainee Research Assistant, Clinsearch Healthcare Solutions; 3 Medical student, Inter Cultural Program for Freshman, Med (ICPF-Med), Fachhochschule des Mittelstands (FHM); 4 Department of Dentistry, King Edward Memorial Hospital, Mumbai, India; 5 Asst. Prof of Pharmacology, Bharati Vidyapeeth Deemed University Dental College & Hospital, Navi Mumbai; 6 Research Associate, Clinsearch Healthcare Solutions; 7 Asst. Director, Clinical Research, Institute of Infectious Diseases (IID), Pune

**Keywords:** burnout, burnout syndrome, medical practitioners, ambi, bcsq-12, chronic stress, physician health, mental health, india

## Abstract

**Background and objectives:**

Excessive and prolonged work-related stress has always been a cause for burnout among healthcare professionals. This has led to emotional, mental, and physical exhaustion. This survey was conducted to assess the burnout among medical practitioners using the abbreviated Maslach Burnout Inventory (aMBI) and Burnout Clinical Subtype Questionnaire (BCSQ-12) scales.

**Materials and methods:**

A cross-sectional survey was conducted among 482 registered medical practitioners across India. A questionnaire consisting of 25 socio-demographic and occupational questions related to aMBI and BCSQ-12 scales was used to assess the burnout. The distribution of responses for each variable was examined using frequencies and percentages among the subgroups to find out the burnout levels of various components of the scales.

**Results:**

High burnout levels were uniformly recorded for the entire population. For the aMBI, 45.02% (n = 217) and 65.98% (n = 318) of the participants scored high on the emotional exhaustion and depersonalization scales, respectively, whereas 87.14% (n = 420) scored low on the personal accomplishment scale and 62.86% (n = 303) and 11.41% (n = 55) had medium and low scores on the satisfaction with the medical practice scale. The BCSQ-12 scale showed the mean values of 15.89, 11.56, and 10.28 on a scale of 28 for overload, lack of development, and neglect subtypes, respectively, whereas, satisfaction with the financial compensation item showed a mean value of 3.79 on a scale of seven. All these values indicate high levels of burnout.

**Conclusion:**

The results suggest high levels of burnout in all domains of aMBI and BCSQ-12 scales in all the occupational and socio-demographic groups of medical practitioners and warrant immediate actions to address this issue.

## Introduction

Chronic stress in the work environment is one of the leading factors for burnout syndrome and could have a deleterious effect on health. Burnout is defined as a feeling of hopelessness and inability in carrying out one's job effectively [1]. It is a psychological and physical response which may arise when the employees are exposed to a stressful working environment involving high expectations, inadequate resources, and low compensation. This can be seen when an individual fails to control the work-related stress effectively. Burnout has three primary components—exhaustion (feeling of not being able to give any more of oneself to work), cynicism (distancing behaviour towards work, customers, and co-workers), and inefficiency (feelings of inadequacy and incompetence when performing tasks at work) [2]. Due to its nature and our inability to detect it easily, burnout can be a serious problem in the medical profession. It needs to be addressed promptly, especially in India where the burden of healthcare of the population rests on the shoulders of a limited number of doctors.

Burnout has been studied among practicing doctors in many parts of the world including the USA [3-4], European countries [5-6], and Latin America [7], while very few studies are being reported from India [8-9]. In a study conducted in the USA in 2012, 7288 physicians completed a survey in which 45.8% of the physicians showed at least one symptom of burnout. There was an approximately 10% higher level of burnout and 17% higher level of dissatisfaction as compared to the general population in this particular study [4]. A high level of emotional exhaustion, depersonalization, and low personal accomplishments were reported among the nurses and respiratory therapists working in the intensive care unit (ICU) in the US [3]. Though the burnout in the medical field seems to be a universal phenomenon, it is not well documented in the literature. This survey was conducted to assess the degree of burnout among the medical practitioners across India and to compare the pattern of burnout amongst the different subgroups based on the gender, age, practice type, qualification, and work experience.

## Materials and methods

This was a questionnaire-based cross-sectional survey to assess the level of burnout among medical practitioners in India. This was part of a larger study used to assess the levels of burnout among healthcare professionals in India. This study was conducted in adherence to good clinical practice (GCP) guidelines and Declaration of Helsinki (DoH). The study documents were reviewed and approved by the Institutional Ethics Committee (IEC) of Bharati Vidyapeeth University Dental College and Hospital (BVDU) (Navi Mumbai, Maharashtra, India).

All participating practitioners had to complete a 25-item self-administered questionnaire. The study questionnaire was to be filled by the respondents in a print (paper) format, an electronic format (.pdf) or through an online link. All the participants who consented for the study were requested to complete the printed format and submit them to the researchers. The electronic form was designed as a portable document format (.pdf) and was sent to the potential respondents by e-mail. The electronic forms were downloaded and filled by the responders and returned via postal or courier service or emailed back as scanned copies. Additionally, an online link to fill the form was also made available to the respondents who wished to submit their forms online. All the electronic and print forms were checked for completeness, and any deficiency or discrepancies were resolved by the respondents via mail, short message service (SMS), or telephonic communication.

Convenient sampling was used for data collection, and participants from medical colleges, hospitals, clinics, seminars, continuing medical education (CME) seminars, and conferences from various parts of the country were included in the study. Records of medical organizations like the Indian Medical Association (IMA) (Mumbai and Vadodara branches) and the Association of Medical Consultants (AMC) were used to collect the contact information of the participants. Emails were sent to various physicians explaining the scope and objective of the study. In addition to an invitation to participate, a link to the survey that contained a description of the study was provided. The participation was purely voluntary. The duration of the study was from December 2014 to June 2016.

An attempt was made to reach out to a large number of medical practitioners across India; however, the majority of the participants who responded were from the state of Maharashtra. We were able to reach 9691 practitioners of whom a total of 482 responded. The respondents were from Maharashtra (n = 408), Karnataka (n = 19), Gujarat (n = 10), Haryana (n = 13), Rajasthan (n = 8), Jammu and Kashmir (n = 1), Tamil Nadu (n = 8), Andhra Pradesh (n = 1), and Kerala (n=2). Some participants had not specified their state (n = 12). Medical practitioners of either gender, who had a Bachelor of Medicine and Bachelor of Surgery (MBBS) degree or above, from any state or union territory of India and had registered with their respective medical council were contacted for the survey. Additionally, only those respondents who had a minimum experience of five years either in academics, practice, or both after their basic qualification was completed qualified for the study. Any respondents who did not meet the above criteria were excluded from the analysis.

The demographic profile, academic qualifications, and the work profile of respondents were captured. Burnout was assessed using two scales—the abbreviated MBI and the BCSQ-12. Both these scales are validated and have been used in earlier studies to assess the burnout among medical practitioners, students, and healthcare workers [10-15]. The aMBI consists of three main domains of burnout. The emotional exhaustion subscale (items 3, 4, and 7) assesses the feelings of being emotionally overextended and exhausted by one's work. The depersonalization subscale (items 2, 5, and 8) measures an unfeeling and impersonal response towards the recipients of one's service, care, or treatment. The personal accomplishment subscale (items 1, 6, and 9) assesses the feelings of competence and successful achievement in one's work with the people. An additional fourth subscale, career satisfaction in medicine (items 10, 11, and 12) has been added to the original inventory to assess the satisfaction with one’s work [13, 16]. For this study, the participants had to indicate their degree of agreement with each of the statements presented according to a Likert-type scale with the seven response options scored from zero (totally disagree) to six (totally agree). The burnout scores of zero to six were considered low, seven to 12 were considered moderate, and 13–18 were considered high for each domain as recorded by the earlier studies [17-19].

Similarly, the BCSQ-12 scale consists of three dimensions for burnout. It contains 12 items equally distributed between the three dimensions, 'overload' (e.g. "I overlook my own needs to fulfil work demands"), 'lack of development' (e.g. "My work doesn't offer me opportunities to develop my abilities"), and 'neglect' (e.g. "When things at work don't turn out as well as they should, I stop trying"). The ‘overload’ dimension consists of items 1, 4, 7, and 10, the ‘lack of development’ dimension consists of items 2, 5, 8, and 11, and the ‘neglect’ dimension consists of the items 3, 6, 9, and 12. The participants had to indicate their degree of agreement with each of the statements presented according to the Likert-type scale with seven response options, scored from one (totally disagree) to seven (totally agree) as used in the earlier studies [20]. The higher scores indicated the higher levels of burnout.

Additionally, we also collected data for satisfaction with respect to 'adequate financial compensation in their profession' using a seven-point Likert scale scored from one (totally disagree) to seven (totally agree).

The sample was divided into various subgroups on the basis of age, gender, qualification, and job profile. The subgroups based on age were 25–35 years (mean experience of 6.87 years), 36–50 (mean experience of 16.6 years), and 51 and above (mean experience of 34.75 years). The two subgroups based on qualification were graduates and postgraduates, and the three subgroups based on job profile were the clinicians, academicians, and those engaged in both.

The completed questionnaires were coded and the data was tabulated prior to analysis. The distribution of the responses for each variable was examined using frequencies and percentages. Descriptive statistics were presented for the scores of questionnaire domains in the subgroups based on the age, gender, qualification, and work experience. Mean scores were calculated for the individual subscales of BCSQ-12 and MBI to find out the prevalence of burnout levels in each of these components.

## Results

Out of the total participants, 66.80% (n = 322) were males, 33.20% (n = 160) were females. The distribution of the participants in the various subgroups of age, experience, qualification, and job profile is provided in Table 1.

**Table 1:** Distribution of the participants according to the four domains of the MBI Burnout scores of 0–6 were considered low, 7–12 were considered moderate, and 13–18 were considered high for each domain of the MBI scale.

Groups	Level of Burnout	MBI Domain Emotional Exhaustion	MBI Domain Depersonalization	MBI Domain Personal Accomplishment	MBI Domain Satisfaction with Medicine
Total participants (n=482)	High	45.02% (n=217)	65.98% (n=318)	2.07% (n=10)	25.73% (n=124)
	Medium	32.78% (n=158)	25.73% (n=124)	10.79% (n=52)	62.86% (n=303)
	Low	22.41% (n=108)	8.30% (n=40)	87.14% (n=420)	11.41% (n=55)
Qualification					
Graduate (n=115)	High	39.13% (n=45)	60% (n=69)	2.61% (n=3)	20% (n=23)
	Medium	40% (n=46)	32.17% (n=37)	9.57% (n=11)	70.43% (n=81)
	Low	20.87% (n=24)	7.83% (n=9)	88.70% (n=102)	9.57% (n=11)
Postgraduate (n=367)	High	46.87% (n=172)	66.22% (n=249)	2.18% (n=8)	27.52% (n=101)
	Medium	30.25% (n=111)	23.14% (n=87)	11.17% (n=41)	60.49% (n=222)
	Low	22.89% (n=84)	8.24% (n=31)	86.65% (n=318)	11.99% (n=44)
Gender					
Males (n=322)	High	44.10% (n=142)	65.53% (n=211)	1.55% (n=5)	25.47% (n=82)
	Medium	33.54% (n=108)	27.64% (n=89)	10.25% (n=33)	62.11% (n=200)
	Low	22.36% (n=72)	6.83% (n=22)	88.20% (n=284)	12.42% (n=40)
Females (n=160)	High	46.88% (n=75)	66.88% (n=107)	3.13% (n=5)	26.25% (n=42)
	Medium	30.63% (n=49)	21.88% (n=35)	11.88% (n=19)	64.38% (n=103)
	Low	22.5% (n=36)	11.25% (n=18)	85% (n=136)	9.38% (n=15)
Profession					
Academics (n=35)	High	34.29% (n=12)	28.57% (n=10)	8.57% (n=3)	8.57% (n=3)
	Medium	17.14% (n=6)	31.43% (n=11)	5.71% (n=2)	48.57% (n=17)
	Low	48.57% (n=17)	40% (n=14)	85.71% (n=30)	42.86% (n=15)
Clinical practice (n=278)	High	46.76% (n=130)	70.86% (n=197)	1.80% (n=5)	24.82% (n=69)
	Medium	33.81% (n=94)	24.46% (n=68)	11.87% (n=33)	65.83% (n=183)
	Low	19.42% (n=54)	4.68% (n=13)	86.33% (n=240)	9.35% (n=26)
Both the above (n=169)	High	44.38% (n=75)	65.68% (n=111)	7.69% (n=13)	1.18% (n=2)
	Medium	33.73% (n=57)	26.63% (n=45)	8.88% (n=15)	10.06% (n=17)
	Low	21.89% (n=37)	7.69% (n=13)	65.68% (n=111)	88.76% (n=150)
Age groups					
25–​35 years (n=161)	High	33.54% (n=54)	57.76% (n=93)	3.11% (n=5)	23.60% (n=38)
	Medium	38.51% (n=62)	30.43% (n=49)	9.94% (n=16)	64.60% (n=104)
	Low	27.95% (n=45)	11.80% (n=19)	86.96% (n=140)	11.80% (n=19)
36–​50 years (n=160)	High	43.75% (n=70)	68.125% (n=109)	2.5% (n=4)	28.75% (n=46)
	Medium	32.5% (n=52)	24.375% (n=39)	12.5% (n=20)	58.13% (n=93)
	Low	23.75% (n=38)	7.5% (n=12)	85% (n=136)	13.13% (n=21)
>51 years (n=161)	High	57.76% (n=93)	72.05% (n=116)	0.62% (n=1)	24.84% (n=40)
	Medium	26.71% (n=43)	22.36% (n=36)	9.94% (n=16)	65.84% (n=106)
	Low	15.53% (n=25)	5.59% (n=9)	89.44% (n=144)	9.32% (n=15)

**Figure 1:**
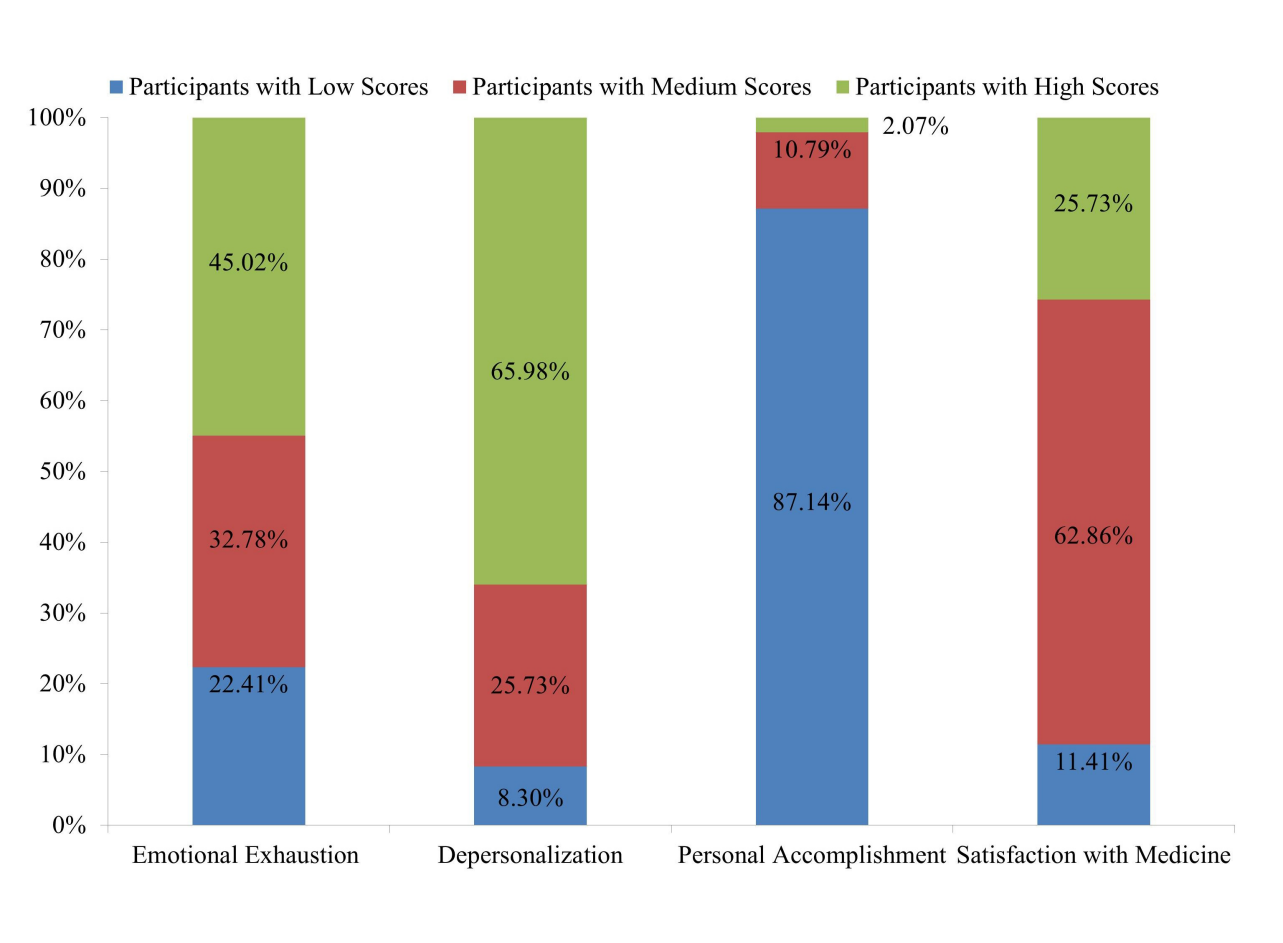
Percentage distribution of participants according to high, medium or low scores obtained on various scales of MBI

The distribution of the aMBI scores is given in Table 1 and Figure 1. For the aMBI emotional exhaustion scale, out of the 482 respondents, 45.02% (n = 217) had high burnout scores. A higher number of postgraduates (46.87%) as compared to graduate doctors (39.13%) were emotionally burnt out. Our study showed that 46.88% (n = 75) of the females showed high burnout which was more than seen in the males (44%, n = 142). A high emotional burnout was seen in more number of participants from the clinicians group and those in the age group of 51 years and above. 

For the aMBI depersonalization scale, out of the 482 respondents, 65.98% (n = 318) had high burnout scores. Yet again, more postgraduates than graduates and more females than males had high scores. Among the various age groups and job profile groups, the clinicians and those above 51 years exhibited the highest number with high depersonalization burnout.

For the aMBI personal accomplishment scale, out of the 482 respondents, about 87.14% (n = 420) had low scores. More number of graduate doctors as compared to postgraduates and more males as compared to females exhibited low personal accomplishment scores. Also, a higher number of participants in the age group of 51 years and above exhibited lower scores. The number of doctors who had low personal accomplishment scores was the highest in those engaged in academics and clinical practice as against the other groups.

Out of the 482 respondents, 25.73% (n = 124) had high scores, 62.86% (n = 303) had medium scores, and 11.41% (n = 55) had low scores on the aMBI satisfaction scale. More postgraduates than graduates had low satisfaction scores and more number of males than females had low scores on the satisfaction scale. In this study, 42.86% of the academicians had low scores whereas 9.35% clinicians and 8.28% participants engaged in both academics and clinical practice had low scores. Amongst the various age groups, the maximum number of participants with low scores was recorded in the age group of 36–50 years.

The results of the BCSQ-12 scores are given in Table 2. For the ‘overload’ dimension of the BCSQ-12, the mean score for the total of 482 respondents was a high score of 15.89 out of a maximum score of 28. The postgraduates exhibited higher mean scores than their graduate counterparts; similarly, the females had higher mean overload scores than the males. The participants who were engaged in clinical practice and academics had higher overload scores. However, the age groups of 25–35 years and 36–50 years had similar mean scores of 16.79 and 16.43, which was much higher than 14.17 seen in the age group of 51 years and above.

**Table 2:** Mean, median, and range for BCSQ-12 scores and the mean scores of 'satisfaction with financial compensation' of the participants belonging to various groups BCSQ-12 has a maximum score of 28 on each of the three components. Satisfaction with financial compensation is an added component that has a maximum score of seven.

	BCSQ-12 Domain			Satisfaction with Financial Compensation
	Overload	Lack of Development	Neglect	
	*Mean/ Median (Range)*	*Mean/ Median (Range)*	*Mean/ Median (Range)*	*Mean/ Median (Range)*
Total participants (n= 482)	15.89/ 16 (4-28)	11.56/ 11 (4-28)	10.28/ 10 (4-25)	3.79/ 4 (1-7)
Qualification				
Graduate (n=115)	15.43/ 16 (4-28)	11.3/ 11 (4-28)	10.12/ 11 (4-20)	3.75/ 4 (1-7)
Postgraduate (n=367)	16.04/ 16 (4-28)	11.65/ 11 (4-28)	10.33/ 10 (4-25)	3.8 / 4 (1-7)
Gender				
Males (n=322)	15.55/ 16 (4-28)	11.31/ 11 (4-28)	10.18/ 10 (4-25)	3.89/ 4 (1-7)
Females (n=160)	16.58/ 17 (4-28)	12.06/ 12 (4-25)	10.49/ 11 (4-22)	3.59/ 3 (1-7)
Profession				
Academics (n=35)	15.25/ 15 (4-28)	12.22/ 12 (4-24)	11.14/ 12 (4-20)	3.97/ 5 (1-7)
Clinical practice (n=278)	15.49/ 16 (4-28)	11.22/ 11 (4-28)	10.13/ 10 (4-25)	3.82/ 4 (1-7)
Both the above (n=169)	16.69/ 19 (4-28)	11.99/ 11 (4-28)	10.35/ 10 (4-22)	3.71/ 4 (1-7)
Age groups				
25–​35 years (n=161)	16.79/ 17 (4-28)	11.95/ 12 (4-25)	10.37/ 10 (4-20)	3.22/ 3 (1-7)
36–​50 years (n=160)	16.43/ 17 (4-28)	11.83/ 11.5 (4-28)	10.53/ 11 (4-25)	4.06 / 4.5 (1-7)
>51 years (n=161)	14.17/ 15 (4-28)	10.92/ 11 (4-28)	9.94/ 10 (4-22)	4.1/ 5 (1-7)

For the ‘lack of development’ dimension of the BCSQ-12, the mean score for the total of 482 respondents was a high score of 11.56 out of 28. The postgraduate doctors had higher mean scores than the graduates. Also, the females exhibited higher mean scores than the males. Higher mean scores were seen in the academicians followed by those engaged in both clinical practice and academics as compared to those engaged in clinical practice only. The lack of development scores decreased as the age group increased.

The ‘neglect’ dimension of the BCSQ-12 had a mean score of 10.28 out of 28 for the total of 482 respondents. All the gender, qualification, and job profile groups exhibited similar neglect mean scores in the range of 10.12–10.49. For the age group of 51 years and above, the mean score was 9.94, much lower than 10.53 and 10.37 as seen in the age groups of 36–50 years and 25–35 years, respectively.

Item 13, which was added to measure the satisfaction with financial compensation, showed a low mean value of 3.79 on a scale of one to seven for the whole sample. This low value indicates that there is a low level of satisfaction with the financial compensation in the medical profession. The graduates had a lower mean score than their postgraduate counterparts. The males exhibited lower mean satisfaction scores than the females with respect to financial compensation. From among the job profile groups, the academicians showed the highest satisfaction scores. The age group 51 and above had the highest satisfaction scores followed by the 36–50 years age group, and the lowest score was seen in the 25–35 age group. This indicates that the satisfaction with financial compensation increases with age.

## Discussion

Various scales in the past have been used to identify burnout such as Oldenburg Burnout Inventory (OLBI), Copenhagen Burnout Inventory (CBI), MBI, and the BCSQ. Out of these inventories, we decided to use the MBI for its high validity and the short time needed to complete the questionnaire [21]. Additionally, we also used the short version of the BCSQ, which has been validated and successfully used in previous studies [20]. Both these scales helped us to record the different components of burnout providing a wider picture.

This study has strived to assess the amount of burnout among the medical practitioners from various fields with a wide range of clinical experience across India. Large sections of the sample population had high burnout scores on the emotional exhaustion and depersonalization components and low scores on the personal accomplishment and satisfaction components of the aMBI scale. Thus, all the four components of the aMBI scale indicated high levels of burnout in this study. Similarly, high mean values were obtained for all the three components of the BCSQ-12 scale. The satisfaction with the financial compensation item indicated that the majority of the sample population found the financial compensation to be inadequate for the amount of work and effort put in. Thus, both these scales indicate a high prevalence of burnout among the medical practitioners across India.

Our study shows that 45.02% (n = 217) and 65.98% (n = 318) of the participants scored high on the emotional exhaustion and depersonalization scales respectively, 87.14% (n = 420) scored low on the personal accomplishment scale and 62.86% (n = 303) and 11.41% (n = 55) had medium and low scores on the satisfaction with the medical practice scale. Such high prevalence of burnout in Indian medical practitioners is similar to the results of studies done on burnout among the medical practitioners in other countries. Also, the results of the study are similar to the earlier studies done on the intensive care unit (ICU) doctors in India [22]. Previous studies done in the US showed higher rates of burnout in the medical practitioners as compared to the general population. The high rates of emotional exhaustion recorded in this study are similar to previous studies conducted on the oncologists in the US [23]. The burnout levels in this study are higher than those found in the studies conducted on European doctors. The European General Practice Research Network (EGPRN), in their study on the burnout among family doctors in Europe, concludes that burnout is a common problem, with high levels affecting two-thirds of the respondents in the study. Overall, 41% of the participants reported high levels of emotional exhaustion, 35% reported depersonalization, and 32% had low feelings of personal achievement, which are lower than the results seen in this study [6].

This study shows a high prevalence of burnout among females as compared to the males. Earlier studies on the French intensivists have reported a higher level of burnout among the females as compared to their male counterparts [24]. A high burnout is seen more commonly in females in India, probably because of higher expectations in the domestic settings, causing a discrepancy in their work-life balance.

Additionally, there seems to be a rise in the burnout levels with increase in the respondents' age and experience. This finding is similar to earlier findings where there was a rise in burnout with an increase in experience [25]. The increase in age would mean more amount of time spent working in the profession, compounding the effect of disturbed work-life balance, resulting in higher burnout levels.

The burnout scores of the postgraduate doctors were marginally higher than their graduate counterparts on various components of the two scales. This shows that burnout increases with increase in specialty training and practice. This was expected as the number of specialists is very limited, and hence they are subjected to more working hours. Also, the nature of their jobs is very demanding. When academicians, clinicians, and those engaged in both roles were compared, it was seen that a majority of the participants among the academicians had lower scores on emotional exhaustion and depersonalization components. The group of participants engaged in both clinical practice and academics showed a drastically high number of participants who scored low in the satisfaction component of the aMBI scale. The mean scores on the BCSQ-12 scale were similar for the three groups. The overall burnout among the health care academicians was higher than those reported in the previous studies [26]. Since the academicians work in an institute-based setup they devote more time to teaching and have limited working hours in the clinic as compared to the practitioners. This explains why they probably fare better on emotional exhaustion, depersonalization, and personal accomplishment components. They score lower on the satisfaction with medical profession component probably because of lower salaries and limited growth opportunities as compared to private practice.

Similar findings of high burnout were also seen in dental practitioners who were part of a larger study to assess burnout in the health care profession. This may indicate that burnout is prevalent not only among medical practitioners, but also other professionals across the health care system in India and needs urgent attention. 

High levels of burnout are probably seen due to the poor doctor-patient ratio in India. The number of specialty training programs are limited, resulting in a lot of work pressure on a few specialists. The intense patient workload with limited doctors results in long working hours. This leaves them with minimal time for family life and recreation. Also seen in this study is that most of the practitioners are unhappy with the remuneration offered by the profession. About three-quarters of the patients are not covered by insurance and they pay for their own treatment. Many patients are unable to provide adequate compensation due to the financial restraints. Also, the medical fees here are lower than the other developed countries. These multiple factors have led to intense dissatisfaction among the medical community, which is a cause of great concern.

Clinical burnout has been associated with multiple adverse effects. Burnout is an occupational health issue of a psychosocial nature and is one of the most important work-related problems in today’s society. Burnout may lead to somatic symptoms like interpersonal problems, insomnia, irritability, and suicidal ideation and may closely resemble a psychological mood disorder known as dysthymia [27]. Burnout has been linked to risk factors of cardiovascular disease (CVD) [28]. It has been associated with high levels of cholesterol, glucose, triglycerides, uric acid, and marginally, with the electrocardiography (ECG) abnormalities. The participants who have scored high on intense burnout also demonstrated significantly higher low-density lipoprotein (LDL) level. Additionally, scores on burnout plus listlessness were significantly associated with glucose and negatively with diastolic blood pressure [28]. Burnout has also been linked to a higher risk of type II diabetes [29]. Thus, burnout is a major cause of concern for the physical and psychological well-being of the medical professionals.

In an era of cut-throat competition and doctors’ unrealistic expectations of perfectionism, strategies for overcoming stress-inducing practices must be incorporated. Early detection and early treatment is the key to tackle the problem effectively. The utilization of better ergonomic practices would limit the potential for progression of stress and thereby the development of burnout. Also, stress management should be a part of the training in the work environment. Stress management programs should be implemented at the institute level. Studies show that introducing stress management courses was associated with a substantial reduction in the rate of malpractice claims in hospitals where such courses were introduced as compared to control hospitals where no such courses were conducted [30]. Also, an individual approach along with an institutional approach would be more effective in improving physician well-being. In addition to stress management at the workplace, doctors should be encouraged to take breaks from their daily activities and work. A well-balanced professional and personal life will go a long way in reducing burnout in the medical profession.

The sample size of 482 has provided us with a good insight into the burnout levels in the population of medical practitioners in India and this is the merit of this study. However, one drawback is that the data is skewed to more respondents from the state of Maharashtra and does not truly represent the entire nation. Thus, further studies are required to get more accurate data and confirm the findings of this study.

## Conclusions

This study found a high prevalence of burnout among medical professionals. Burnout among medical practitioners can be dealt with support from medical bodies and organisations, by maintaining a good work-life balance, and obtaining an understanding from the patients of their problems.
